# Comparison of ^18^F SPECT with PET in myocardial imaging: A realistic thorax-cardiac phantom study

**DOI:** 10.1186/1471-2385-6-5

**Published:** 2006-10-31

**Authors:** Karin Knešaurek, Josef Machac

**Affiliations:** 1 Division of Nuclear Medicine, The Mount Sinai Medical Center, New York, USA

## Abstract

**Background:**

Positron emission tomography (PET) imaging with fluorine-18 (^18^F) Fluorodeoxyglucose (FDG) and flow tracer such as Rubidium-82 (^82^Rb) is an established method for evaluating an ischemic but viable myocardium. However, the high cost of PET imaging restricts its wider clinical use. Therefore, less expensive ^18^F FDG single photon emission computed tomography (SPECT) imaging has been considered as an alternative to ^18^F FDG PET imaging. The purpose of the work is to compare SPECT with PET in myocardial perfusion/viability imaging.

**Methods:**

A nonuniform RH-2 thorax-heart phantom was used in the SPECT and PET acquisitions. Three inserts, 3 cm, 2 cm and 1 cm in diameter, were placed in the left ventricular (LV) wall to simulate infarcts. The phantom acquisition was performed sequentially with 7.4 MBq of ^18^F and 22.2 MBq of Technetium-99m (^99m^Tc) in the SPECT study and with 7.4 MBq of ^18^F and 370 MBq of ^82^Rb in the PET study. SPECT and PET data were processed using standard reconstruction software provided by vendors. Circumferential profiles of the short-axis slices, the contrast and viability of the inserts were used to evaluate the SPECT and PET images.

**Results:**

The contrast for 3 cm, 2 cm and 1 cm inserts were for ^18^F PET data, 1.0 ± 0.01, 0.67 ± 0.02 and 0.25 ± 0.01, respectively. For ^82^Rb PET data, the corresponding contrast values were 0.61 ± 0.02, 0.37 ± 0.02 and 0.19 ± 0.01, respectively. For ^18^F SPECT the contrast values were, 0.31 ± 0.03 and 0.20 ± 0.05 for 3 cm and 2 cm inserts, respectively. For ^99m^Tc SPECT the contrast values were, 0.63 ± 0.04 and 0.24 ± 0.05 for 3 cm and 2 cm inserts respectively. In SPECT, the 1 cm insert was not detectable. In the SPECT study, all three inserts were falsely diagnosed as "viable", while in the PET study, only the 1 cm insert was diagnosed falsely "viable".

**Conclusion:**

For smaller defects the ^99m^Tc/^18^F SPECT imaging cannot entirely replace the more expensive ^82^Rb/^18^F PET for myocardial perfusion/viability imaging, due to poorer image spatial resolution and poorer defect contrast.

## Background

Fluorine-18 (^18^F) Fluorodeoxyglucose (FDG) single photon emission computed tomography (SPECT) imaging has been considered as an alternative to ^18^F FDG positron emission tomography (PET) imaging for evaluating injured but viable myocardium [1,2]. SPECT ^18^F imaging is sometimes performed as simultaneous Technetium-99m sestamibi (^99m^Tc MIBI)/^18^F FDG perfusion/viability myocardial studies [3,4]. The main limitations of this technique are poor resolution of the ^18^F and ^99m^Tc SPECT images and, when acquired simultaneously, degradation of the ^99m^Tc MIBI images due to ^18^F downscatter to the ^99m^Tc window. The ^18^F images are also influenced by high septal penetration and poor sensitivity, due to the limited stopping power of the detector crystal in standard gamma cameras [5]. In PET centres without a cyclotron, sequential Rubidium-82 (^82^Rb) and ^18^F FDG perfusion/viability myocardial imaging are the most appropriate, because of the availability of both isotopes [6-10]. ^82^Rb is a potassium analog that, like Thalium-201, is extracted by living cells. It is produced from a commercially available, FDA approved strontium-82 generator, which must be replenished 13 times a year. The short half-life of ^82^Rb allows repeated acquisitions every 10 minutes. Neither, ^18^F FDG or ^82^Rb require expensive on-site cyclotrons, as are needed with ^13^N ammonia and ^15^O oxygen. Also, most health care providers reimburse for ^82^Rb perfusion imaging, and more recently for ^18^F FDG viability myocardial imaging.

The objective of our work was to compare ^18^F SPECT with PET in myocardial perfusion/viability imaging in the classification of simulated myocardial defects for "viability" according to commonly applied criteria.

## Methods

**Figure 1:**
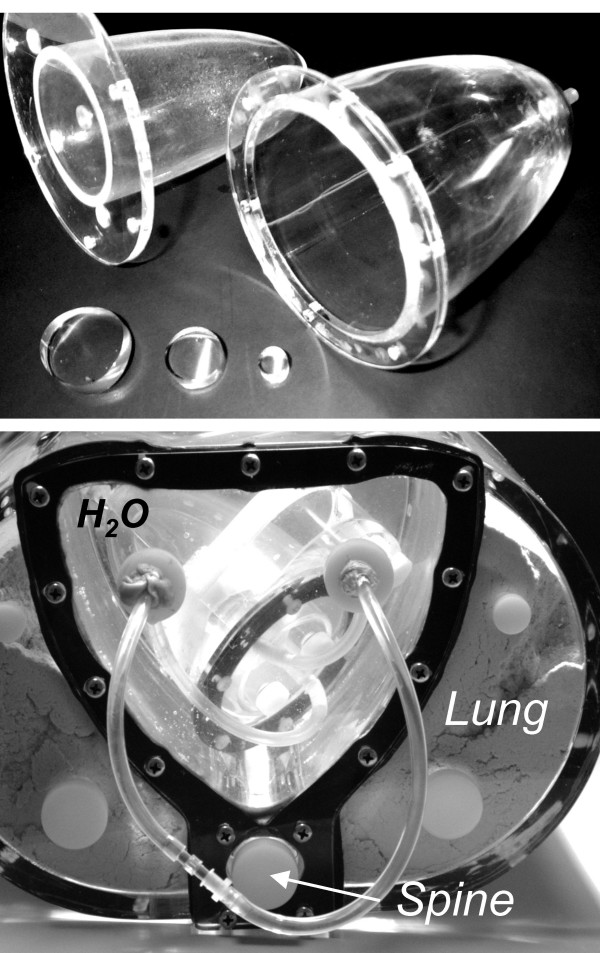
**RH-2 thorax-heart phantom**. A) In a left ventricular wall three inserts, 1 cm, 2 cm and 3 cm in diameter were placed in the same short-axis plane. B) Cardiac phantom is placed in water. Left ventricular wall is connected by Teflon tubes to remote filling system. Teflon rod simulates spine and sawdust simulates lungs.

**Figure 2:**
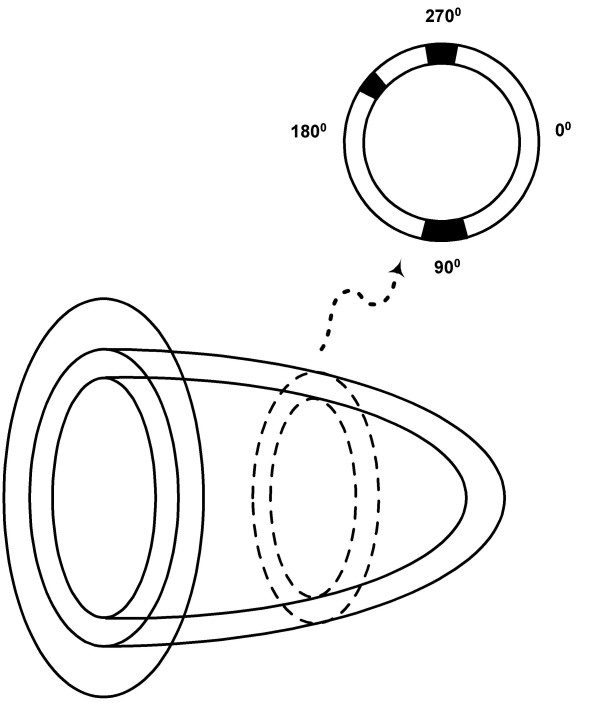
**Position of inserts**. The 3 cm insert was placed at 90 degrees, 1 cm insert at 200 degrees and 2 cm insert at 270 degrees.

A nonuniform RH-2 thorax-heart phantom (Kyoto Scientific Speciment Co., LTD, Kyoto, Japan) (Fig. 1) was used in SPECT and PET acquisitions. Three inserts, 3 cm, 2 cm and 1 cm in diameter, were placed in the left ventricular (LV) wall to simulate small transmural infarcts (Fig. 2).

SPECT acquisitions were performed on a dual headed system (T22 gamma camera, SMV, Twinsburg, Ohio) with ultra-high-energy (UHE) collimator. The energy resolution of the system at 140 keV is 9.8%, and at 511 keV is 7.8%. The thickness of the NaI(Tl) crystal is 0.9525 cm (3/8"). A step-and-shoot mode was used for SPECT acquisitions, with a 64 × 64 acquisition and processing frame matrix size and zoom of 1.5. The time per frame was 40 sec. The 64 frames were acquired over 360°. The radius of rotation for the phantom study was 21 cm, which is also a typical radius for our clinical SPECT acquisitions. The pixel size in the acquisition and reconstructed images was 6.08 mm. SPECT data were processed using standard reconstruction software based on a filtered backprojection method. A Butterworth filter of 5th order and cut-off frequency of 0.25 cycles/pixel was used in the reconstruction for all studies. Neither attenuation nor scatter correction was performed.

The UHE parallel-hole collimator, used for the ^18^F SPECT imaging, has hexagonal holes 3.4 mm in diameter and 65.0 mm in length, with a septal thickness of 3.0 mm. The collimator cores are mounted in an all-lead frame designed for 511 keV imaging. Planar resolution as a function of distance for the UHE collimator, measured in air with a ^99m^Tc point source was FWHM = 3.9 mm, 10.6 mm, and 14.0 mm for 0 cm, 10 cm and 15 cm distance, respectively. The same planar resolution measured with an ^18^F point source gave FWHM = 5.3 mm, 13.9 mm, and 18.2 mm for 0 cm, 10 cm and 15 cm distance, respectively [11]. The standard deviation was less than 0.5 mm in all measurements and was obtained from three measurements. This shows that planar resolution for the UHE collimator is better for ^99m^Tc than for ^18^F. Septal penetration for the UHE collimator is 6.5% and for the low-energy-high-resolution collimator is 0.50% [5]. The quoted septal penetration values were calculated for only single septi. Actual septal penetration in clinical situations may account for 30%–50% of detected events [3].

A GE ADVANCE (General Electric Medical Systems, Milwaukee, WI) PET system was used in 2D mode for PET acquisitions. The system has 18 detector rings and 12,096 bismuth germanate (BGO) 4 × 8 × 30 mm crystals. In 2D mode, the system uses a tungsten collimator 1 × 120 mm in size. The axial field of view is 15.2 cm. It is covered by 35 image planes. The axial sampling interval is 4.25 mm. The transaxial field of view is 55.0 cm. The coincidence window width is 12.5 ns and energy window is 300–650 keV. All 2D PET acquisitions were performed in high sensitivity (HS) mode. The resolution of our PET system was measured using an ^18^F line source and for HS mode, FWHM = 4.44 ± 0.04 mm in the tangential direction and FWHM = 4.85 ± 0.08 mm in the radial direction. The PET images were reconstructed using a filtered backprojection reconstruction method and Hanning smoothing filter with an 0.5 cycles/pixel cutoff. The matrix size was 128 × 128 and the pixel size was 4.29 mm. Attenuation correction using an 8-min transmission scan was applied in the PET studies. Also, Bergstrom [12] scatter correction, provided by the vendor, was applied.

All phantom acquisitions were single-isotope studies in order to avoid down-scatter. The SPECT study was performed with 7.4 MBq of ^18^F and 22.2 MBq of ^99m^Tc and the PET study with 7.4 MBq of ^18^F and 370 MBq of ^82^Rb. These activities were injected into the phantom LV wall, which with the three inserts had a volume of 140 ml. Consequently, the radioactivity concentrations in the LV wall were 52.9 kBq/ml for ^18^F, 158.6 kBq/ml for ^99m^Tc and 2.64 MBq/ml for ^82^Rb.

**Figure 3:**
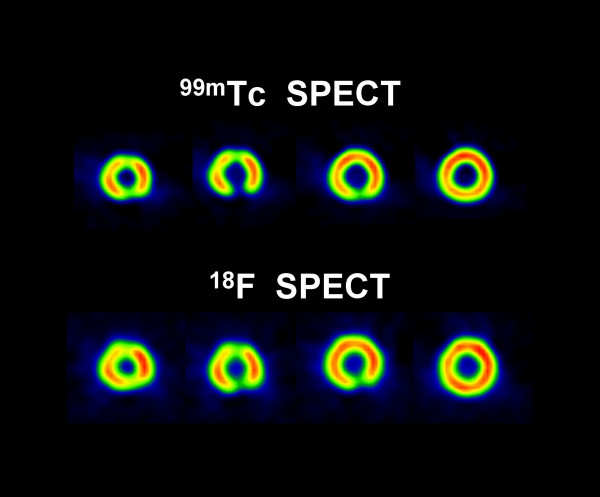
**Short-axis slices**. Short-axis slices in the ^99m^Tc/^18^F SPECT cardiac phantom study.

**Figure 4:**
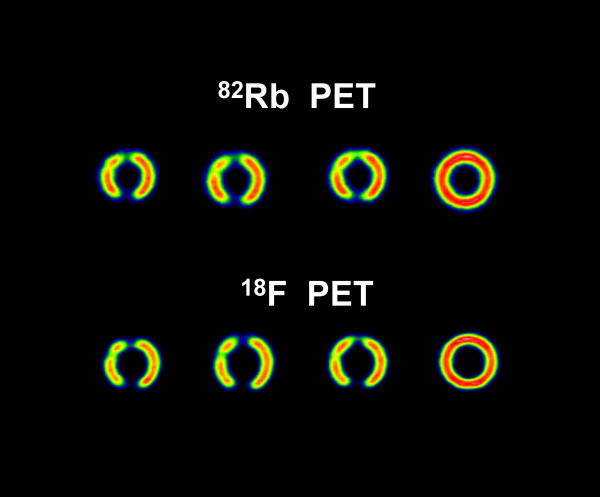
**Short-axis slices**. Short-axis slices in the ^82^Rb/^18^F PET cardiac phantom study.

Circumferential profiles of the short-axis slices (figs. 3 and 4), the contrast, and the defect severity of the inserts were used to evaluate the SPECT and PET images. The contrast value was calculated as a ratio C = (A-B)/(A+B), where A and B are the average activities in region-of-interest (ROI) covering the normal and cold areas, respectively. In the SPECT study, 3 × 3 pixel (region-of-interest) ROIs covering 333 mm^2 ^were used, and in the PET study 4 × 4 pixel ROIs covering 294 mm^2 ^were used. The different size ROIs in SPECT and PET were used to approximately cover the same area, although the PET ROI was slightly smaller. In all studies, three adjacent short-axis slices were used, and their average contrast value and standard deviation were calculated.

Two criteria were used to determine "viability" or "nonviability" [13-24]. First, for each isotope images, i.e., ^99m^Tc, ^82^Rb and ^18^F images, if the minimum value in the lesion (MIN) was less than 50% of the maximum LV value (MAX), the lesion was considered to be nonviable. In addition to the above mentioned criterion for nonvaibility, in the SPECT study, the minimum lesion value in the ^18^F image (MIN(^18^F)) should be equal to or less than the same corresponding minimum lesion value in the ^99m^Tc image (MIN(^99m^Tc)). In the PET study, the minimum lesion value in the ^18^F image should be equal to or less than the same the minimum lesion value in the ^82^Rb image (MIN(^82^Rb)). If the MIN(^18^F) is greater than the same lesion MIN(^99m^Tc) or MIN(^82^Rb), for SPECT or PET study, respectively, then the lesion is considered viable (mismatch pattern).

By definition, the inserts, not containing any activity, were considered as a "nonviable" gold standard.

## Results

**Figure 5:**
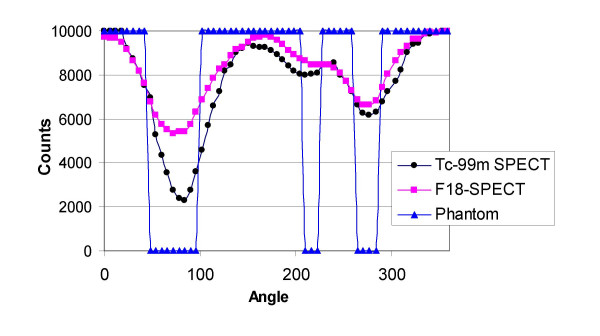
**Circumferential profiles**. Circumferential profiles from short-axis slices shown in fig. 3.

Figures 3 and 4 show short axis slices from the ^99m^Tc/^18^F SPECT study and from the ^82^Rb/^18^F PET study, respectively. In the SPECT study (Fig. 3) the ^99m^Tc images have better resolution and contrast than the corresponding ^18^F images [5,11]. However, in the PET study (Fig. 4) the situation is the opposite, i.e. the ^18^F images have better resolution and contrast compared with the corresponding ^82^Rb images. Comparing the SPECT with PET images (Fig. 3 and Fig. 4) one can see that PET images have much better resolution and, except for the ^99m^Tc 3 cm insert, the contrast values are better than in the SPECT images, regardless of the isotope used. For ^18^F PET data the contrast for 3 cm, 2 cm and 1 cm inserts was 1.0 ± 0.01, 0.67 ± 0.02 and 0.25 ± 0.01, respectively. For ^82^Rb PET data the corresponding contrast values were 0.61 ± 0.02, 0.37 ± 0.02, and 0.19 ± 0.01, respectively. For ^18^F SPECT the contrast values were, 0.31 ± 0.03 and 0.20 ± 0.05 for 3 cm and 2 cm inserts respectively. For ^99m^Tc SPECT the contrast values were, 0.63 ± 0.04 and 0.24 ± 0.05 for 3 cm and 2 cm inserts respectively. In the SPECT study the 1 cm insert was not visually detectable (Fig. 3 and Fig. 5).

The total number of counts was 2.5 times higher in the PET ^18^F study vs. the SPECT ^18^F study. The total number of counts in the PET study was calculated as total prompt counts minus total delays (randoms), and they include scatter events.

**Figure 6:**
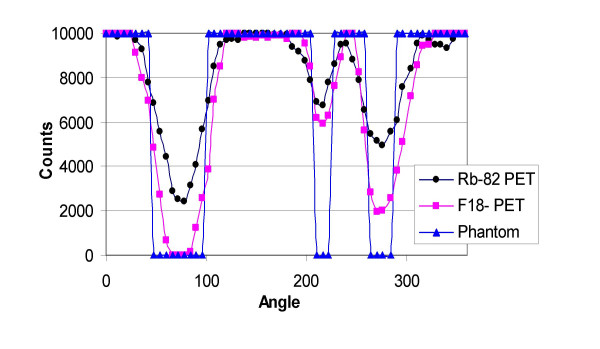
**Circumferential profiles**. Circumferential profiles from short-axis slices shown in fig. 4.

Figures 5 and 6 show circumferential profiles from the short-axis slices shown in fig. 3 and fig. 4, respectively, normalized to 10,000 counts. Normalization provided easier calculations of nonviability/viability of the insert areas, using the criteria described above. Table 1 gives minimum values in the insert areas as percentage of the maximum, i.e. 10,000 values. Based on the criteria described in the methods section, nonviability/viability for the inserts were calculated and presented in Table 2.

**Table 1:** COMPARISON OF SPECT ^99m^Tc/^18^F AND PET ^82^Rb/^18^F IN CARDIAC PHANTOM STUDY*

	**1 cm insert**	**2 cm insert**	**3 cm insert**
^18^F SPECT	85%	66%	54%
^99m^Tc SPECT	80%	62%	23%
^18^F PET	60%	20%	0%
^82^Rb PET	68%	50%	24%

* Percentage of maximum values

**Table 2:** Clinical diagnosis

	**1 cm insert**	**2 cm insert**	**3 cm insert**
^99m^Tc/^18^F SPECT	VIABLE	VIABLE	VIABLE
^82^Rb/^18^F PET	VIABLE	NONVIABLE	NONVIABLE

Based on the ^99m^Tc/^18^F dual-isotope criteria used (Tables 1 and 2), SPECT failed, for all three inserts, to provide the correct result (nonviable), while the ^82^Rb/^18^F PET study correctly identified the two larger nonviable lesions. The region of the 1 cm insert was falsely diagnosed as viable by using the criteria described in the methods section, because MIN(^18^F) was 60% of the MAX(^18^F) value in the LV wall. However, the 1 cm insert area is clearly visible in ^18^F and R-82 PET images (Fig. 4) and their corresponding circumferential profiles (Fig. 6). On the contrary, in the ^99m^Tc/^18^F SPECT study, the 1 cm insert was not clearly visible in the reconstructed images (Fig. 3) and circumferential profiles (Fig. 5).

## Discussion

The results show that PET study provides images of better resolution and sensitivity than SPECT study. This results in better contrast for cold lesions created by the inserts and better determination of viability/nonviability using standard criteria. The differences in resolution and sensitivity between PET and SPECT studies are significant. The difference in sensitivity between the PET ^18^F study vs. the SPECT ^18^F study is a factor of 2.5. The difference in resolution is less pronounced, but sill present. For ^18^F SPECT the FWHM = 5.3 mm, while for ^18^F PET FWHM = 4.44 ± 0.04 mm in the tangential direction and FWHM = 4.85 ± 0.08 mm in the radial direction. These differences in resolution and sensitivity results in significantly better PET images comparing with the SPECT images in the study (Fig. 3, 4, 5, Fig. 6).

Using commonly applied criteria [13-24], SPECT failed for all three inserts to provide the accurate diagnosis of nonviability, which is significant drawback. While 1 cm lesion may not be clinically important, as discussed below, failure to properly classify 2 cm and 3 cm lesions may be a problem for wider ^18^F SPECT clinical application. On the other hand, PET correctly diagnosed 2 cm and 3 cm lesions as nonviable and failed only for 1 cm insert to provide accurate diagnosis because MIN(^18^F) was 60% of the MAX(^18^F) value in the LV wall. One way to improve PET diagnostics accuracy is to raise the threshold from 50% to 60% for our PET system. But, 1 cm lesion is not of clinical importance because question of viability usually apply to large segment or multisegment region. However, in the PET study 1 cm insert was clearly visible in both the ^18^F and ^82^Rb images, while in the ^99m^Tc/^18^F SPECT study a 1 cm insert was not clearly visible in the images and circumferential profiles (Fig.3, 4, 5, Fig. 6).

In addition, an important difference between the SPECT and PET images of the phantom was that ^18^F SPECT images were of inferior quality compared with SPECT ^99m^Tc images, while ^18^F PET images were better in quality than corresponding ^82^Rb PET images. Consequently, for SPECT, the defects were seen as mismatches, while in PET they tended to be seen as reverse mismatches.

The reason for this is that the gamma camera was optimized for ^99m^Tc 140 keV photon imaging. Resolution properties for ^18^F imaging are better on the PET system than on the gamma camera. Septal penetration is also a significant problem in gamma camera ^18^F imaging. In our high count phantom studies sensitivity was not the main issue, but in a clinical situation that can also be a problem for standard gamma cameras. For a 0.9525 cm (3/8") thick NaI(Tl) crystal 73% of ^18^F 511 keV photons do not interact, 15% interact through Compton scatter and only 12% through the photoelectric effect, which is the mode most desirable for good detection. This means that for ^18^F 511 keV photons, standard gamma cameras have very poor sensitivity. Doubling the thickness of the NaI(Tl) crystal to 1.905 cm (3/4") will increase the number of photoelectric interactions to 26%, Compton scatter to 20%, and will decrease the number of noninteracting photons to 54%. But, still this represents relatively poor sensitivity, especially when compared with ^99m^Tc 140 keV photons interacting with a 0.9525 cm (3/8") thick NaI(Tl) crystal: 88% photoelectric effect, 2% Compton scatter and only 10 % no interaction.

^82^Rb PET images have poorer resolution and contrast when compared with ^18^F images due to a higher ^82^Rb positron maximum energy of 3.35 MeV (emitted 83% of the time) and 2.57 MeV (emitted 12% of the time). In comparison, ^18^F positron maximum energy is only 0.64 MeV. Higher positron energy results in higher average distance positron travel in medium prior to annihilation, 2.40 mm for ^82^Rb vs. 0.35 mm for ^18^F.

This study examined the effect of system resolution on the classification of small intramural defects as "viable" or "nonviable" by commonly used criteria [13-24]. Over the last two decades, a variety of criteria have been used to define viability, few of which are exactly the same. Nevertheless, the criteria used here are some of the most commonly used. Use of alternate criteria, i.e., 60% threshold [22,23], or other arbitrary or empirical criteria would result in similar, if not identical, conclusions. In practice, questions of viability usually apply to large segmental and multisegmental regions of dysfunction, hypoperfusion and hypometabolism [13-24], since small perfusion (function) defects have little impact on LV functional properties. Small defects of 2 cm or less would not be considered clinically for questions of viability. However, perceived false mismatches at the edges of defects several cm in extent may result in misclassification of significant portions of the myocardium as viable in SPECT studies, as would modest defects of 2–4 cm. To a lesser extent, false negatives of viability may occur in ^82^Rb/^18^F PET studies.

A similar phantom comparison between SPECT and a dedicated PET system [1] concluded that by using an appropriate threshold to define a defect, SPECT can accurately measure defect size similarly to the manner of PET. Also, the same group recently published the results of their clinical comparison of SPECT with PET in the assessment of myocardial viability [4], and concluded that SPECT provided similar information to that of PET and MRI. The phantom used was very similar to our phantom, the PET system was the same and, as in our study, motion and background effects were not taken onto account. These studies recognize already mentioned advantages of PET over SPECT, which has higher spatial resolution, higher count sensitivity and routine use of attenuation and scatter correction in PET, but not in SPECT. However, the difference in the UHE collimator design and properties, the difference in the gamma camera crystal thickens, our 0.9525 cm (3/8") vs. 1.6 cm (5/8") thick NaI(Tl) crystal in these studies [1,4], a difference in acquisition (360 degree in our studies vs. 180 degree in these studies) and a difference in evaluation of SPECT and PET data lead us to a slightly different conclusion.

## Conclusion

The results of this phantom study suggest that for smaller defects, the ^99m^Tc/^18^F SPECT imaging cannot entirely replace the more expensive ^82^Rb/^18^F PET for myocardial perfusion/viability imaging, due to poorer image spatial resolution and poorer defect contrast.

## Competing interests

The author(s) declare that they have no competing interests.

## Authors' contributions

Both authors, KK and JM, have made substantial contributions in acquisition, processing and analysing of data, and writing the manuscript.

## Pre-publication history

The pre-publication history for this paper can be accessed here:


